# Digital Response During the COVID-19 Pandemic in Saudi Arabia

**DOI:** 10.2196/19338

**Published:** 2020-09-01

**Authors:** Marwah Hassounah, Hafsa Raheel, Mohammed Alhefzi

**Affiliations:** 1 Prince Sattam Chair for Epidemiology and Public Health Research, Department of Family and Community Medicine College of Medicine King Saud University Riyadh Saudi Arabia; 2 Preventive Medicine and Clinical Informatics King Faisal Medical City for Southern Regions Abha Saudi Arabia; 3 Saudi Association for Health Informatics Riyadh Saudi Arabia

**Keywords:** digital response, COVID-19, Saudi Arabia, digital health, containment, public health, pandemic, prevention

## Abstract

**Background:**

The first case of COVID-19 in Saudi Arabia was confirmed on March 3, 2020. Saudi Arabia, like many other countries worldwide, implemented lockdown of most public and private services in response to the pandemic and established population movement restrictions nationwide. With the implementation of these strict mitigation regulations, technology and digital solutions have enabled the provision of essential services.

**Objective:**

The aim of this paper is to highlight how Saudi Arabia has used digital technology during the COVID-19 pandemic in the domains of public health, health care services, education, telecommunication, commerce, and risk communication.

**Methods:**

We documented the use of digital technology in Saudi Arabia during the pandemic using publicly available official announcements, press briefings and releases, news clips, published data, peer-reviewed literature, and professional discussions.

**Results:**

Saudi Arabia’s government and private sectors combined developed and launched approximately 19 apps and platforms that serve public health functions and provide health care services. A detailed account of each is provided. Education processes continued using an established electronic learning infrastructure with a promising direction toward wider adoption in the future. Telecommunication companies exhibited smooth collaboration as well as innovative initiatives to support ongoing efforts. Risk communication activities using social media, websites, and SMS text messaging followed best practice guides.

**Conclusions:**

The Saudi Vision 2030 framework, released in 2017, has paved the path for digital transformation. COVID-19 enabled the promotion and testing of this transition. In Saudi Arabia, the use of artificial intelligence in integrating different data sources during future outbreaks could be further explored. Also, decreasing the number of mobile apps and merging their functions could increase and facilitate their use.

## Introduction

The outbreak of SARS-CoV-2, emerging from the markets of Wuhan, led to the COVID-19 pandemic [1,2]. The current population-wide measures of home quarantine that were simultaneously applied worldwide to slow and prevent the spread of COVID-19 are unprecedented. The COVID-19 pandemic has caused disruption of daily services due to the community-wide mitigation measures taken by many countries. Due to the low likelihood of obtaining a vaccine in the near future, global efforts have vastly focused on social distancing and complete city and state lockdowns in many instances as the only solutions to contain the pandemic [3]. These mitigation measures have necessitated the use of technology to maintain functions in all aspects of life.

The global experiences with the H1N1 influenza pandemic in 2009 [4] and Ebola virus in 2014 clearly indicated that timely and appropriate technology usage played a considerable role in controlling these pandemics [5-7]. A cloud computing tool for data collection and integration for confirmed cases of Middle East respiratory syndrome coronavirus (MERS-CoV), a GPS-based risk assessment tool [8], and Google Maps usage for the geographical representation of MERS-CoV cases worldwide are examples of the technological methods used to control the outbreak of Middle East respiratory syndrome (MERS) [8]. By establishing a national electronic surveillance system [9,10], Saudi Arabia also contributed to the global data pool of MERS-CoV information.

During the current COVID-19 pandemic, Saudi Arabia has been proactive in implementing disease containment measures and working to meet the community’s needs and demands in a very short time [3]. It is currently estimated that 30,260,000 people in Saudi Arabia (89% of the population) use the internet, 96% of the population uses smartphones [11], and the majority of the population now has access to smartphones, laptop computers, desktop computers, and tablets; therefore, digital service provision is much easier than in the past and has aided the mitigation efforts established by the government.

Keeping in view the importance of quick and timely digital data sharing for policy actions, which is also emphasized by the World Health Organization (WHO) [12], our aim in this paper is to highlight how Saudi Arabia has used digital technology during the COVID-19 pandemic.

## Methods

The authors documented Saudi Arabia’s experience using publicly available official announcements, press briefings and releases, news clips, published data, peer-reviewed literature, and professional discussions. The searched information sources were in both English and Arabic languages. A literature search was conducted from March 20 to June 20, 2020. Each author collected, examined, and synthesized information on a designated sector; then, all the authors consolidated, discussed, and agreed on the final findings. The inclusion criteria for information were that the information depicted a prominent event during the COVID-19 pandemic response, included technology or digitalization, and was specific to Saudi Arabia. The findings are mainly presented in narrative form.

## Results

**Figure 1:**
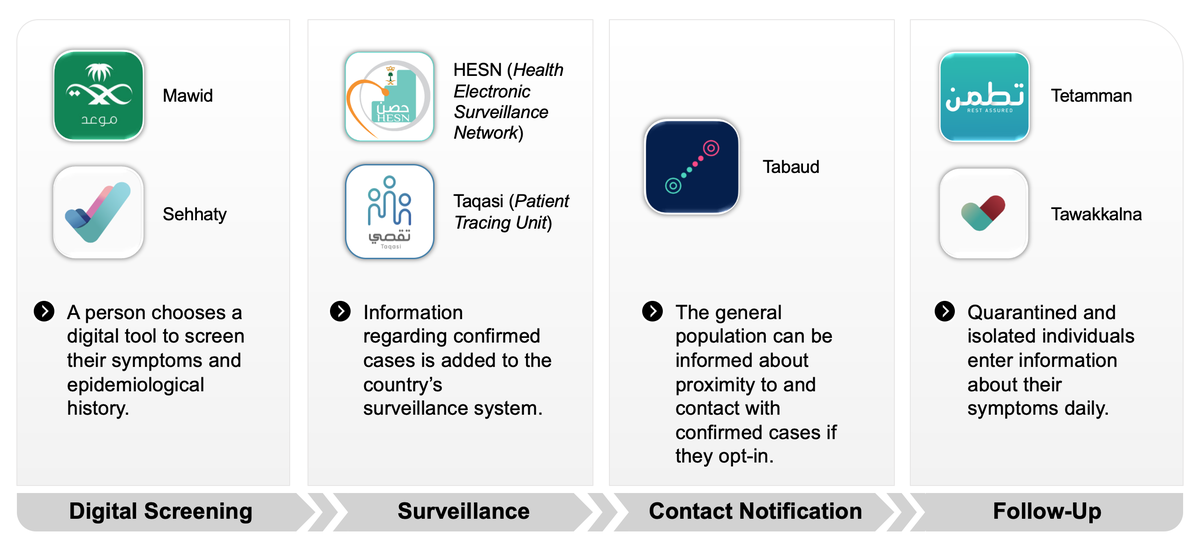
Examples of digital apps available for various health care domains during the COVID-19 pandemic in Saudi Arabia.

Figure 1 and Table 1 visualize and summarize some of the applications and platforms used for various health sectors during the COVID-19 pandemic in Saudi Arabia.

**Table 1:** Summary of telehealth applications available in Saudi Arabia during the COVID-19 pandemic.

Name	Type	Short description	Provider
Sehha [13]	Smartphone app (iOS/Android)	Teleconsultation (synchronous live video chat, nonsynchronous SMS text messaging).	Ministry of Health
Mawid [14]	Smartphone app (iOS/Android), web-based application	Symptom checker/appointment gateway to all Saudi Ministry of Health Services. Used as the main channel for the virtual COVID-19 screening/triaging by the Saudi Ministry of Health.	Ministry of Health
Anat [15]	Smartphone app (iOS/Android)	E-prescription^a^ gateway; licensure of all health care professionals is checked with the Saudi Commission for Health Specialties.	Ministry of Health
Wasfaty [16]	Web-based	The official e-prescription gateway provided by the Ministry of Health.	Ministry of Health
Asefni [17]	Smartphone app (iOS/Android)	GPS-enabled requests for emergency services nationwide.	Saudi Red Crescent Authority
Cura [18]	Smartphone app (iOS/Android)	Teleconsultations (synchronous live video chat, nonsynchronous SMS text messaging, more specific subspecialties/for-profit).	Private
MayaClinic [19]	Smartphone app (iOS/Android)	Teleconsultations (nonsynchronous text messaging with health care providers).	Private
Nala [20]	Smartphone app (iOS/Android)	Teleconsultations (artificial intelligence-enabled chatbot provides decision support for the public, acts as an appointment gateway and nonsynchronous messaging with health care providers).	Private
Labayh [21]	Smartphone app (iOS/Android)	Teleconsultations (mainly provides psychology sessions and mental health services).	Private
80/20 Lifestyle [22]	Smartphone app (iOS/Android)	Remote patient engagement and lifestyle change recommendations.	Private
Virtual Medical Academy [23]	Web-based interactive academy	Videoconferencing events targeted to health care professionals.	Private
SCFHS Webinars [24]	Web-based seminars	Offers support services to health care professionals under their programs (Daem for residents and Emtenan for all health care professionals).	Saudi Commission for Health Specialties

^a^e-prescription: electronic prescription.

### Health Sector Digitalization

Amid the COVID-19 pandemic, the Saudi Ministry of Health has implemented multiple informatics tools to provide public health information for individuals as well as the community.

### Public Health Informatics Tools

In 2018, the Ministry of Health launched a national central health care appointment gateway through a mobile app and web-based application called Mawid, which translates to “Appointment” [14] (Figure 1). Soon after, in August 2019, the Sehhaty (“My Health”) app was launched in the pursuit of a wide range of health promotional campaigns that target healthy lifestyles, using gamification and community-wide challenges [25]. However, both apps were updated to respond to the COVID-19 pandemic by introducing a symptom checker to enable people who suspect they have COVID-19 to directly book appointments at dedicated COVID-19 clinics [26] and drive-through mass testing locations around the Kingdom [27].

For COVID-19 surveillance, the Health Electronic Surveillance Network (HESN) has been mainly used as a reliable source of data for all COVID-19 laboratory tests in the Kingdom. The HESN serves as a national communicable disease surveillance platform. It was launched in 2012 and piloted during the largest public health event in Saudi Arabia: the annual pilgrimage season, or the Hajj [28]. Moreover, the Patient Tracing Unit (Taqasi) platform was implemented in March 2020 for the COVID-19 pandemic. Its purpose is to enhance and manage contact tracing around the Kingdom based on the laboratory results generated from the HESN.

Locally published preventative and clinical guidelines give directions for home isolation with documented daily follow-ups and for tracking symptoms for mild cases and contacts. To provide these functions, the National Health Emergency Operation Centre launched a smartphone app, Tetamman, which translates as “Rest Assured” [29]. In May 2020, the Ministry of Health announced that the Tetamman app will also be associated with a smart bracelet for individuals returning from abroad as well as those who are isolated in their homes [30].

Contact tracing has been termed as an essential epidemiologic tool for containing the COVID-19 outbreak and enforcing future plans for lifting lockdown safely. To achieve this, the Saudi Data and Artificial Intelligence Authority (SDAIA) released two smartphone apps. The first is Tawakkalna, a GPS-enabled app to monitor and restrict individuals’ movement during curfew hours with the capacity to issue permits for exceptions. The second app, Tabaud, whose name means “Distancing” [31], sends deidentified data to people who came in close contact with confirmed cases of COVID-19. The app follows the international Google and Apple guidelines on data privacy. 

### Health Care Delivery

The Saudi Ministry of Health (MOH), as the main health care provider in the Kingdom of Saudi Arabia, is looked upon as the main source of authentic and reliable health information for the Saudi population. Other channels of health care delivery include the Ministry of Defense, university teaching hospitals, and the private sector. Similarly, tertiary, secondary, and primary care facilities provide health care to both nationals and nonnationals. In 2011, the Saudi MOH agreed upon a vision to improve the standards, equitability, availability, and quality of health care in the Kingdom of Saudi Arabia by the use of electronic communication and information technology in this sector. The Vision 2030 National Transformation Program [32] health care strategic objectives for the years 2018 to 2020 aimed to increase access to care, improve quality, and promote the prevention of health risks. It highlights electronic health (eHealth) as an essential enabler to the health care transformation; hence, it tasks the National Health Information Centre with creating multisectoral coherent eHealth services. During the aforementioned community-wide measures to combat the spread of COVID-19, the government of Saudi Arabia and the private health care sector activated existing digital health solutions and produced new ones.

The MOH Call (937) Service Center was established to answer inquiries related to COVID-19. Moreover, one hospital initiated a remotely controlled robot for rounding and monitoring of intensive care unit patients [33].

For hospitals that had teleconsultation apps in place before COVID-19, whether well-established or in pilot phases, some activated their apps to serve patients who do not require in-person hospital visits, such as King Saud Medical City in the public sector and Dr Sulaiman Al Habib Medical Group in the private sector. The group messaging app WhatsApp remains the preferred messaging app in the Kingdom [11]; some hospitals and medical cities in the Eastern Region, such as Qatif Central Hospital and its primary care centers, initiated WhatsApp numbers to help patients register their medication refill requests, arrange for remote routine follow-ups, and inquire regarding their laboratory results.

Periods of pandemics have been shown to cause a surge in stress related to fear of the unknown and isolation. Hence, the literature highlights the need for establishment of psychological support to communities during such periods. To achieve this objective, the National Centre for Mental Health Promotion collaborated with the developers of a local mobile counselling app, Labayh, to provide free sessions for people experiencing anxiety and panic symptoms in the current situation [21]. In another instance, the Saudi Commission for Health Specialties (SCFHS) nationally launched a set of mental health support services for all health care professionals in the Kingdom under its Emtenan initiative as well as for residents in training (Daem) [24]. The SCFHS not only called its registered health care professionals but also sent them SMS text messages, enquiring about their safety and advising them to keep safe.

In the area of telepharmacy, the MOH and other tertiary health care facilities sent medications to patients’ homes via courier companies or established telepharmacy services [15]. Furthermore, the MOH sent out SMS text reminders to all health care providers with active professional registration to use its electronic prescription (e-prescription) services in collaboration with private sector pharmacies. One example is the Anat mobile app [15], which enables providers to directly electronically prescribe medications to patients by credentialed and licensed providers. Other apps that have been active in recent years in Saudi Arabia are Wasfaty [16], translated as “My Prescription,” which is the official gateway for e-prescriptions under the Ministry of Health’s free services, and the Sehha [13] mobile teleconsultation app, which can provide patients with e-prescriptions via SMS text message following a medical consultation with a physician. Private telehealth services such as Cura [18] and Maya Clinic [19] are offering similar services either freely or with modest charges to support their COVID-19 efforts.

With the success of telemedicine services, King Salman bin Abdulaziz Al Saud of Saudi Arabia issued a royal decree to amend health professionals’ practice regulations to allow telemedicine use for diagnostic and management purposes from the workplace and at home. This royal order also directs all relevant sectors to amend their regulations to accommodate this change [34].

### Educational Sector Digitalization

According to Saudi national statistics [35], approximately 1,353,619 students are enrolled in 28 governmental and 34 private higher education institutes. Moreover, there are approximately 5000 schools in the Kingdom that provide secondary level education; these include both public and private sector institutions.

Electronic learning (e-learning) is not new in the Kingdom. Its first decade (1990-2000) in Saudi Arabia’s education system was supported well by the evolution of computer technology and the World Wide Web [36]. By 2002, Saudi Arabia had established a national school e-learning platform with tailored electronic lessons [36]. The following years witnessed expansion and enhancement of e-learning in collaboration with international partners [36]. In 2017, as part of Vision 2030, the Ministry of Education (MOE) established the National Center for e-Learning [37]. This center serves to supervise and support eLearning in Saudi Arabia. The current COVID-19 pandemic poses immense challenges to maintaining continuity of educational services across the Kingdom. This challenge was most evident in the health educational sector due to the absence of a standard and unified method of eLearning and because educational methods depend on patient interactions.

It is already known that major universities in the Kingdom such as King Saud University, Taibah University, King Khalid University, Qassim University, Islamic University of Madinah, Al-Baha University, and King Abdul-Aziz University are the most active e-learning university partners in the Kingdom. However, higher education institutions were challenged by the COVID-19 situation to continue tutoring and assessment of technical skills [38]. Hence, universities offered different methods of e-learning support depending on the course requirements and interim assessment needs [39].

Later, the Minister of Education congratulated higher educational institutes on their successful shift to distance learning since the COVID-19 outbreak. The universities reported that collectively, 1.2 million users were conducting 107,000 hours of web-based learning in more than 7600 virtual classes [40]. The MOE also directed higher education students and faculty to its website “Shams,” an open education resource.

The aforementioned SCFHS is Saudi Arabia’s accreditation and registration body for health care professionals. It offers a series of accredited educational webinars for continuing medical education hours. The SCFHS has collaborated with local and international platforms such as Virtual Medical Academy [23], UpToDate [41], and MDBriefcase [42]. The topics listed therein include a COVID-19 overview, physician burnout, a COVID-19 critical care crash course, and ethical issues during pandemics using COVID-19 as an example [43]. Other nonprofit public and private initiatives have followed the SCFHS’s lead [43,44].

It is worth noting that all educational institutions, including higher education institutions, continued delivery of education during lockdown. Both public and private institutions used various two-way e-learning methods to continue teaching and student learning. This ranged from individual institute-based platforms such as Blackboard and McGraw-Hill Connect to common commercial platforms such as Zoom, Google Class, and FaceTime.

An interesting and unique step taken by the MOE was to shift public school education to its distant learning portals, namely Ein (translated as “Eye”) and Vschool.sa [45]. Ein, which was launched by the Ministry of Education before the COVID-19 outbreak, features a television channel that broadcasts daily lessons based on the national curriculum [39]. During the COVID-19 pandemic, the Ein channel and a corresponding YouTube channel have been redirected to provide live tutoring of all school level subjects and lessons daily from 8:30 AM to 12 PM on weekdays [46]. This great effort was conducted by 127 teachers in 112 subjects. Ein also provides a website through which students can practice lesson exercises and communicate with their teachers [46]. The vschool.sa portal is unique to Saudi Arabia and is a unified learning portal by the Ministry of Education that complements Ein. It provides synchronized web-based tutoring, assessment tools, learning material, and apps for smartphone access [47].

### Telecommunication, Commercial, and Miscellaneous Digital Services

**Figure 2:**
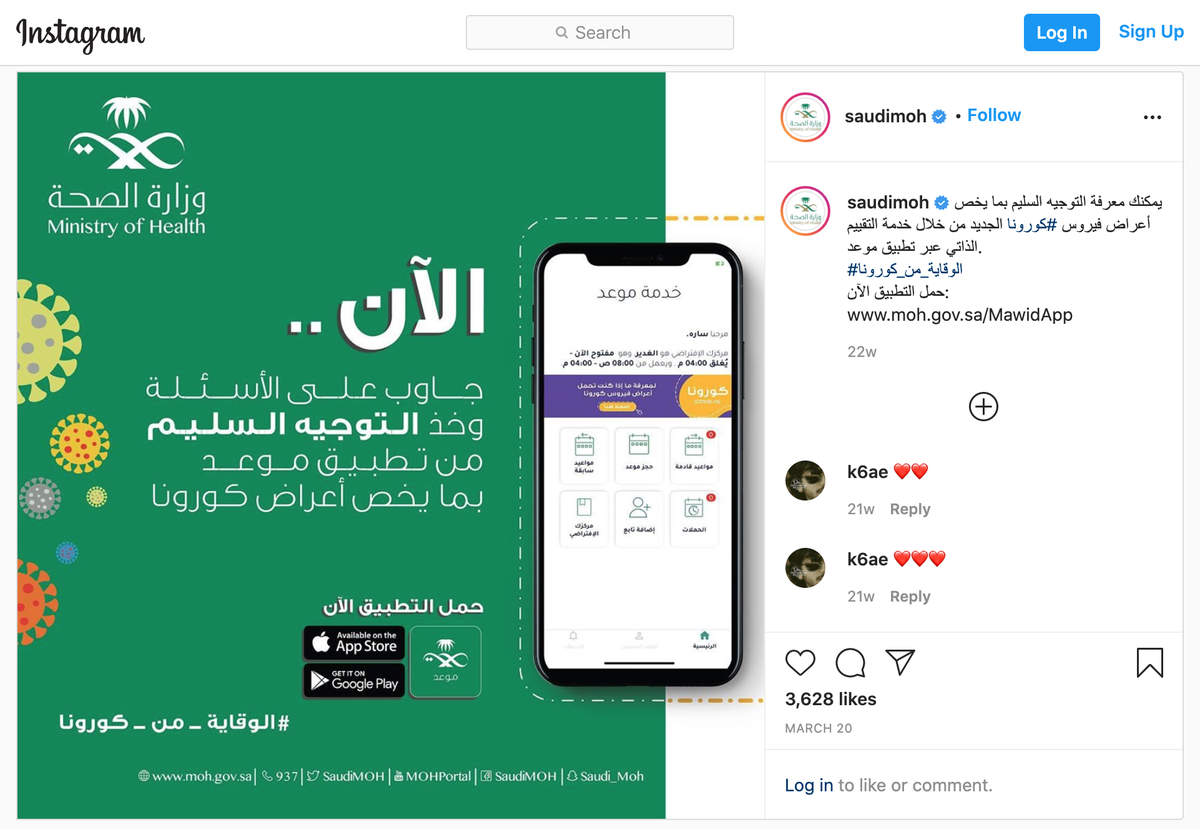
Instagram advertisement for the new symptom checker feature on the Saudi Ministry of Heath’s scheduling mobile app.

The major telecom companies in Saudi Arabia, namely the Saudi Telecom Company (STC), Mobily, and Zain Saudi Arabia, have announced free-of-charge data services to the most used educational platforms as well as health and telehealth applications to facilitate the smooth delivery of e-learning as well as health care delivery during the pandemic. The expected high usage of internet services, which exceeded the current capacity by around 33%, was also supported by the Saudi Communication and Information Technology Commission (CITC), which developed related infrastructure to accommodate the sudden high demand [36]. In an unprecedented move, prior to the COVID-19 pandemic, the CITC had also launched a guide to inform consumers about the trusted available mobile apps that are officially registered within the commission [48]. Internet providers also enabled users to access the Ministry of Health and governmental educational websites without consuming their personal data. When a call is placed on an STC or Mobily mobile number, a voice recording plays that reiterates Ministry of Health messages to help prevent the spread of COVID-19. Telecom companies changed their network names to display a message saying “Stay Home” (Figure 2). All these measures contributed to health education and the awareness drive.

One of the most notable initiatives in Saudi Arabia is “Move to Tech.” This initiative was launched by the Saudi Ministry of Communications and Information Technology on March 10, 2020 [49]. It facilitates the use of current digital tools and the creation of new ones in response to COVID-19. This has increased the use of digital tools in several sectors, but mainly in education, the food industry and health care. Following this initiative, a COVID-19 Hackathon [50] was launched to provide innovative remote and virtual solutions to combat the pandemic.

**Figure 3:**
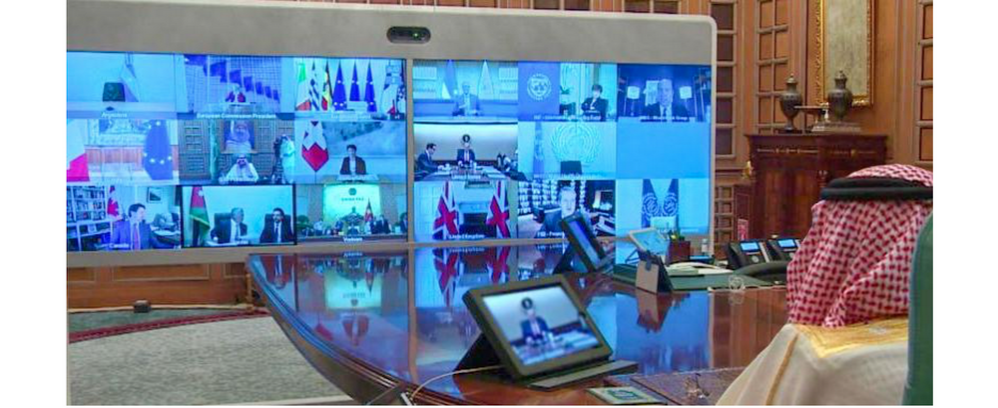
Snapshot of the G20 Extraordinary Virtual Leaders’ Summit on COVID-19, hosted by the Saudi King Salman Bin Abdulaziz, on March 26, 2020.

The G20 International Economic Leaders’ Summit, scheduled in March 2020 and hosted by Saudi Arabia, was required to “go digital” in light of the COVID-19 pandemic. The summit hosted 19 countries, the European Union, and the Central Bank Governors. Saudi Arabia initiated the G20 Extraordinary Virtual Leaders’ Summit on COVID-19 using videoconferencing that was inclusive of all international delegates. It is worth mentioning that the platform used was built and coordinated by the SDAIA (Figure 3) [51,52].

In 2018, the Saudi Red Crescent Authority launched a mobile app, “Asefni,” which translates to “Save Me.” The aim of this app was to facilitate emergency service requests with accurate GPS locations [17]. When strict local curfew and travel restrictions were imposed on all Saudi citizens, the app was updated to provide movement permits during the curfew order for individuals who required essential medical consultations. This permit is issued for each case only after an initial web-based assessment [53]. Similarly, Saudi Public Security launched an online portal, “Tanaqul,” to receive requests for domestic land travel permits between cities for people with extenuating circumstances [54].

Electronic commerce, on the other hand, was a prosperous industry in the Kingdom even before COVID-19. Major retailers had established web-based ordering and home delivery services throughout the major cities for everyday grocery items, home essentials, and furniture. The community-wide quarantine has highlighted the role of these web-based commercial services in aiding the mitigation process.

### Risk Communication Directed at the Public Through Social Media

The year 2011 witnessed a boom of social media and user-generated content in Saudi Arabia [55]. The current social media scene in Saudi Arabia with regard to the percentage of internet users shows that the most preferred and used social media platforms are YouTube, WhatsApp, Facebook, Instagram, and Twitter [11]. In April 2011, the Saudi Ministry of Health joined Twitter and successfully built its audience’s trust over the years until it reached nearly 3 million followers in early 2020 [56,57]. Before the first confirmed case of COVID-19 in Saudi Arabia, the MOH used its website and social media platforms, including Twitter, Facebook, YouTube, Snapchat, Instagram, and TikTok, to distribute health education materials. Different formats were used, such as WhatsApp stickers for proper hygiene. The topics included what COVID-19 is, how it is transmitted, how to prevent getting it, and where it originated. As the pandemic progressed and new scenarios emerged, the literature was modified and expanded to accommodate these changes. It was also translated into other languages, including but not limited to English, Portuguese, French, Russian, Tagalog, Spanish, and Urdu, ensuring a wider spread of relevant information [58]. The MOH and other ministries also used SMS text messages in both English and Arabic to raise awareness and emphasize the practice of precautions. Regular messages were sent to all citizens in different languages.

**Figure 4:**
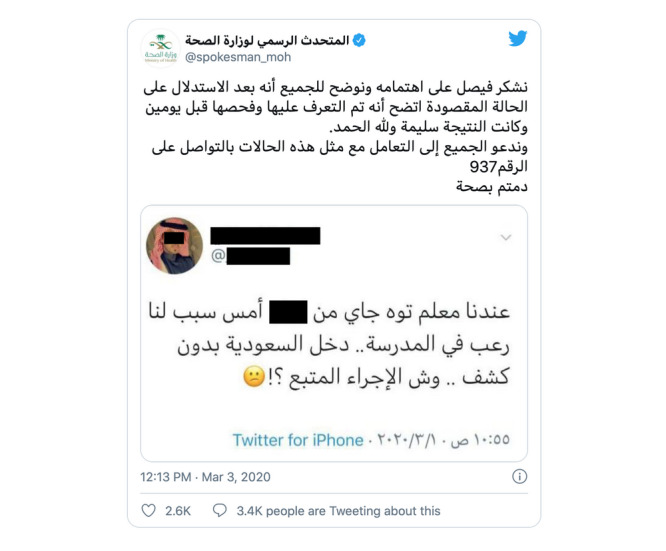
Twitter reply by the Saudi Ministry of Health spokesperson to a tweet by a person who was concerned about a colleague who showed symptoms of respiratory illness after returning from Iran [62].

After the first case of COVID-19 in Saudi Arabia was confirmed on March 3, 2020, the Twitter account of the official spokesperson of the MOH was activated to directly and quickly announce and respond to COVID-19 news [59]. Rumors and misinformation are an expected and organic part of risk communication. According to the WHO, the best practices to address rumors and misinformation in risk and crisis communication include prevention, monitoring, and strategies for approaching a rumor when it occurs [60]. The spokesperson of the MOH incorporated this WHO strategy of dissolving original rumors. On Twitter, he would retweet the rumor with a comment to directly spread the correct information [59]. Figure 4 shows a comment by the MOH spokesperson on a widely distributed tweet by a person who was concerned about a colleague at his school who had just come back from Iran and had shown symptoms of respiratory illness [61]. The spokesperson thanked him for his concern and reassured him that the MOH had reached out to the person of concern and tested him for the virus, and he was found to be negative. He concluded by asking all people to communicate similar concerns to the MOH call center.

The MOH has also collaborated with other government and nongovernment health entities to establish the Prevention Ambassador Initiative. The initiative is a web-based course for the layperson that provides certification in baseline information on COVID-19 to help prevent and control the COVID-19 infodemic [63]. Another effort to contain internet rumors and misinformation is a Saudi Public Prosecution release stating that intentional spread of rumors about COVID-19 or sharing material that causes panic among the public is an electronic crime that can be punished with up to 5 years of imprisonment or a fine of SR 3 million (US $799,888.20) [64].

When curfews were established in a number of major cities, the Center for Government Communication launched a national social media campaign titled *Kollona Masool*, meaning “We Are All Responsible” [65]. The main message of the campaign was that people should stay at home as a patriotic duty to their country and fellow residents. This hashtag was widely used by officials and the general population, and government entities on Twitter changed their cover images to read *Kollona Masool*. At this point, global brands were separating their logos and Twitter user names to emphasize social distancing. Many Saudi government entities and community influencers did the same. Once the lockdown was gradually lifted, this campaign shifted to *Naoodo bi Hathar*, which means “Return Carefully.”

**Figure 5:**
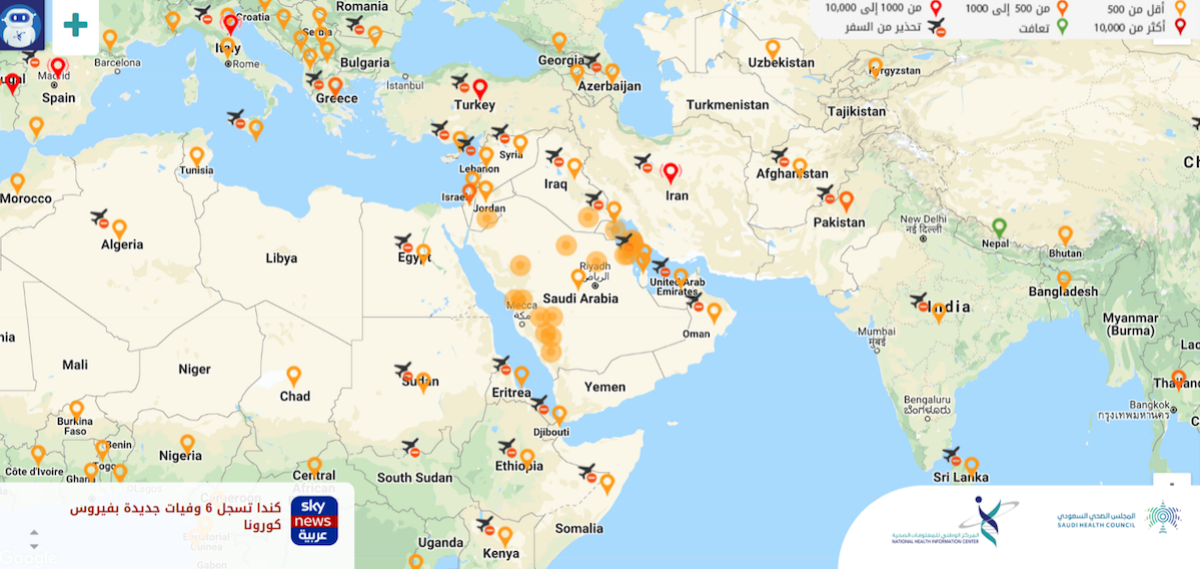
The Arabic language Corona Map provided by the National Health Information Center, under the Saudi Health Council.

Early in the pandemic, the Saudi Health Council and the National Health Information Center started the first Arabic web-based interactive map dedicated to COVID-19 [66] (Figure 5). The map regularly updates travel alerts, confirmed cases, treated cases, deaths, percentage of treated cases, and percentage of deaths. The map is available as an app on the Apple Store [67]. It has an artificial intelligence (AI)–enabled chatbot, “Bashayar,” that offers simple guidance in simple language. It also has a pop-up news headline with the latest official Arabic news on COVID-19 and a dropdown menu with further resources, including educational material, statistics, graphs, sources, data sets, isolation hospitals, statuses of major local and international conferences, and other studies [66].

As more cases were discovered, the National Centre for Disease Prevention and Control (NCDC), also known as Weqaya, launched the COVID-19 hub website, followed by the Ministry of Health’s COVID-19 awareness website [68]. Here, Arabic guidelines and essential information could be found under broad audience categories: community and public, professionals and health care workers, and daily updates [69]. A month into COVID-19 containment measures, the Ministry of Health released a public link to its live local Arabic dashboard, COVID-19 Dashboard: Saudi Arabia [69]. Accessibility to credible facts in a timely manner are core WHO principles of risk communication [60].

## Discussion

### Principal Findings

The ways in which the world has attempted to respond digitally to COVID-19 have surely raised concerns about how the world may be transformed post–COVID-19. On one hand, the need for digital response has been significantly highlighted; on the other hand, major challenges associated with its usage have surfaced [62]. We have already witnessed historical success stories in which countries used technology such as electronic databases and Google Maps to curb the spread of outbreaks.

Even in the current pandemic, several success stories have surfaced in which technology usage helped save lives when global markets shut down and strict curfews were enacted. The United Kingdom, for instance, initiated a COVID-19 symptom tracker app that allows users to enter their symptoms for risk identification, referral, and follow-up [70]. The use of mobile data has been suggested to help identify people at greater risk of travel-related infectious disease and in directing mass screening efforts accordingly. Google and Apple [71] introduced a decision support tool that acts as a location checker and has been implemented in the Tabaud app. Location data gathered from smartphones is used by public health officials to track patterns of movement of quarantined or home-isolated individuals. 

A recent article published in *The Lancet* [72] highlighted the use of AI in curbing COVID-19. Taiwan used AI to improve its national health insurance database and integrate it with its immigration and customs database to create Big Data for analytics and crossmatching of individuals. This system generated alerts during clinical visits based on travel history and clinical symptoms to aid case identification. It also used QR-code scanning and web-based reporting of travel history and health symptoms to classify travelers’ infectious risks based on their flight origins and travel histories for the past 14 days. Persons with low risk (no travel to Level 3 alert areas) were sent a health declaration border pass to their phones via SMS text message for faster immigration clearance; those with higher risk were quarantined at home and tracked through their mobile phones to ensure they remained at home during the incubation period [73]. 

The use of telehealth and chatbots in the United States and Singapore has shown promising results in enabling remote triaging of care and providing rapidly accessible information; these measures enable the provision of care to patients without requiring them to leave their homes [74,75]. 

Saudi Arabia’s digital response to the COVID-19 pandemic is noteworthy. The aforementioned digital tools of public health and health care services are on par with those used worldwide. A few areas still require more exploration, such as the use of AI. It may be desirable to connect all the governmental and nongovernmental apps created during the COVID-19 pandemic to effectively activate interoperability across different technologies. This can lead to the creation of large, continuously updated data sets, which can be later used for diagnosis, management, and policy implementation. 

However, we do recommend decreasing the number of public health mobile apps available for use during a future outbreak. This is to decrease the burden on the end user, avoid confusion, and ensure better adherence. As mentioned previously, there are five applications for COVID-19 symptoms and history screening, follow-up of cases, and contact tracing. Last, it should be ensured that digital location identifiers activated via these applications do not breach privacy and agreed-upon permissions, as both Apple and Google have raised concerns regarding adherence to Health Insurance Portability and Accountability Act (HIPPA) regulations [76].

Wuhan was the first city to implement complete lockdown and initiate the policy of “Suspend Classes Without Stopping Learning.” Lessons from this policy show the importance of having a strong web-based teaching infrastructure, the necessity of building the capacity of teachers, and finding solutions to bridge up the information gap that may occur because of distance teaching [77]. Fortunately, Saudi Arabia already possessed public and private e-learning infrastructure at the time of the COVID-19 pandemic. Saudi universities conducted webinars and training to rapidly increase their faculty’s capacity for e-learning [78]. In one of the MOE’s COVID-19 webinars, a group of education experts found that the COVID-19 experience proved successful in breaking educators’ psychological barriers to use technology and distant learning methods. They also highlighted the future potential of e-learning to enhance web-based question banks and electronic resources, further engage faculty, adopt remote administrative meetings, decrease costs, and improve outcomes [79]. The Minister of Education hinted that distance learning would be made part of the Kingdom’s regular education system [80], as it appears to be the new norm.

The main challenge posed by the COVID-19 pandemic has been the provision of efficient, accurate, and timely information to populations at risk worldwide [81]. The experience of COVID-19 risk communication by the Saudi Ministry of Health was perceived as very useful by 72% and very satisfactory by 74% of a survey population of 3133 Twitter users [82]. Through previous evaluation and improvement efforts, as well as experience with MERS-CoV, the risk communication infrastructure for this pandemic had already been built. Previous literature showed the types and sources of information that people in Saudi Arabia were seeking during the MERS-CoV outbreak. One study showed that 40% of people preferred the internet as a source of information [83].

In 2017, Saudi Arabia underwent a WHO Joint External Evaluation for international health risk assessment, including risk communication. This evaluation documented the use of the MOH web-based social listening tool to monitor rumors and adapt messaging according to the audience [84]. Similarly, Finland used social media messages and email to thematically categorize the community response to COVID-19 and develop recommendations for evidence-based risk communication [85]. Here, we urge the Ministry of Health to document its risk communication experience with COVID-19 for future reference, decision making, and simulation training.

Despite Saudi Arabia’s widespread usage of various technical platforms during the current pandemic, this experience of learning and sharing seems to be ongoing. The community shift toward digital solutions will unravel further challenges and advantages as we continue to control and mitigate the epidemic curve. The implications and impact of this shift are yet to be known and studied. Whether the emerged digital dynamics and new norms among different sectors should be continued after the end of community quarantine remains unanswered. 

### Limitations

We attempted to encompass all digital solutions and tools used during the COVID-19 outbreak in Saudi Arabia up to the time of manuscript revision; however, shortcomings are expected. The COVID-19 pandemic is a rapidly changing scenario with weekly updates. This paper also lists apps but does not evaluate them or check for user experiences. Moreover, the criteria for inclusion in this paper were subjective. The authors attempted to decrease the effect of this subjectivity using discussion and consensus.

### Conclusion

Saudi Arabia has been working to digitally transform many of its sectors since the launch of the national agenda, Vision 2030, in 2017 [32]. The COVID-19 pandemic has expedited this transformation. It has tested the reliability of the country’s digital infrastructure and has highlighted questionable gaps for decision makers. This has been a nationwide trial of Saudi citizens’ acceptance and ability to use and engage with the digitalization of these services and communications. At this point, it is too early to evaluate the unique Saudi experience of population-wide digital solutions. Future research should further explore and analyze the successes and pitfalls, hindrances, and challenges of this digital experience for specific sectors, including institutions, employees, and consumers.

## Abbreviations

AI: artificial intelligence
CITC: Communication and Information Technology Commission
eHealth: electronic health
e-learning: electronic learning
e-prescription: electronic prescription
HESN: Health Electronic Surveillance Network
HIPPA: Health Insurance Portability and Accountability Act
MERS: Middle East respiratory syndrome
MERS-CoV: Middle East respiratory syndrome coronavirus
MOE: Ministry of Education
MOH: Ministry of Health
SCFHS: Saudi Commission for Health Specialties
SDAIA: Saudi Data and Artificial Intelligence Authority
STC: Saudi Telecom Company
WHO: World Health Organization
